# Quantification of glioblastoma mass effect by lateral ventricle displacement

**DOI:** 10.1038/s41598-018-21147-w

**Published:** 2018-02-12

**Authors:** Tyler C. Steed, Jeffrey M. Treiber, Michael G. Brandel, Kunal S. Patel, Anders M. Dale, Bob S. Carter, Clark C. Chen

**Affiliations:** 1 Department of Neurosurgery, Emory University, Atlanta, GA USA; 2 Department of Neurosurgery, Baylor College of Medicine, Houston, TX USA; 3 Department of Neurosurgery, University of California San Diego, La Jolla, CA USA; 4 Department of Neurosurgery, David Geffen School of Medicine, University of California-Los Angeles, Los Angeles, CA USA; 5 Multimodal Imaging Laboratory, University of California San Diego, La Jolla, CA USA; 6 Department of Radiology, University of California San Diego, La Jolla, CA USA; 7 Department of Neurosurgery, Massachusetts General Hospital, Boston, MA USA; 8 Department of Neurosurgery, University of Minnesota, Minneapolis, MN USA

## Abstract

Mass effect has demonstrated prognostic significance for glioblastoma, but is poorly quantified. Here we define and characterize a novel neuroimaging parameter, lateral ventricle displacement (LVd), which quantifies mass effect in glioblastoma patients. LVd is defined as the magnitude of displacement from the center of mass of the lateral ventricle volume in glioblastoma patients relative to that a normal reference brain. Pre-operative MR images from 214 glioblastoma patients from The Cancer Imaging Archive (TCIA) were segmented using iterative probabilistic voxel labeling (IPVL). LVd, contrast enhancing volumes (CEV) and FLAIR hyper-intensity volumes (FHV) were determined. Associations with patient survival and tumor genomics were investigated using data from The Cancer Genome Atlas (TCGA). Glioblastoma patients had significantly higher LVd relative to patients without brain tumors. The variance of LVd was not explained by tumor volume, as defined by CEV or FLAIR. LVd was robustly associated with glioblastoma survival in Cox models which accounted for both age and Karnofsky’s Performance Scale (KPS) (p = 0.006). Glioblastomas with higher LVd demonstrated increased expression of genes associated with tumor proliferation and decreased expression of genes associated with tumor invasion. Our results suggest LVd is a quantitative measure of glioblastoma mass effect and a prognostic imaging biomarker.

## Introduction

Glioblastoma is the most common form of adult brain cancer and remains one of the deadliest of human cancers^1^. Assessment of glioblastoma tumor burden relies on interpretation of magnetic resonance imaging (MRI) in the context of clinical evaluation^2^. The two MR sequences most commonly used in the clinical setting to assess glioblastoma tumor burden include: contrast enhancement (CE) sequences and fluid-attenuated inversion recovery (FLAIR) sequences^3^. Regions of contrast enhancement (CE) displayed on glioblastoma MRI are typically interpreted as the regions of bulk tumor burden^4,5^, whereas regions with high signal intensity on FLAIR, in the absence of radiation therapy, are frequently identified as areas containing invasive tumor or edematous brain.

Because the skull encompasses a fixed volume, growth of neoplastic tissue necessarily results in the displacement of normal cerebrum^6^. This displacement is known as “mass effect” and is a major cause of neurologic injury^7^. While CE volume (CEV) and FLAIR hyper-intensity volumes (FHV) are often used as clinical proxy of glioblastoma tumor burden, these variables provide limited information about the mass effect related to glioblastoma. A key factor that determine the magnitude of the mass effect involves the compliance of the cerebrum. Unfortunately, cerebral compliance cannot be easily determined through imaging findings.

**Figure 1:**
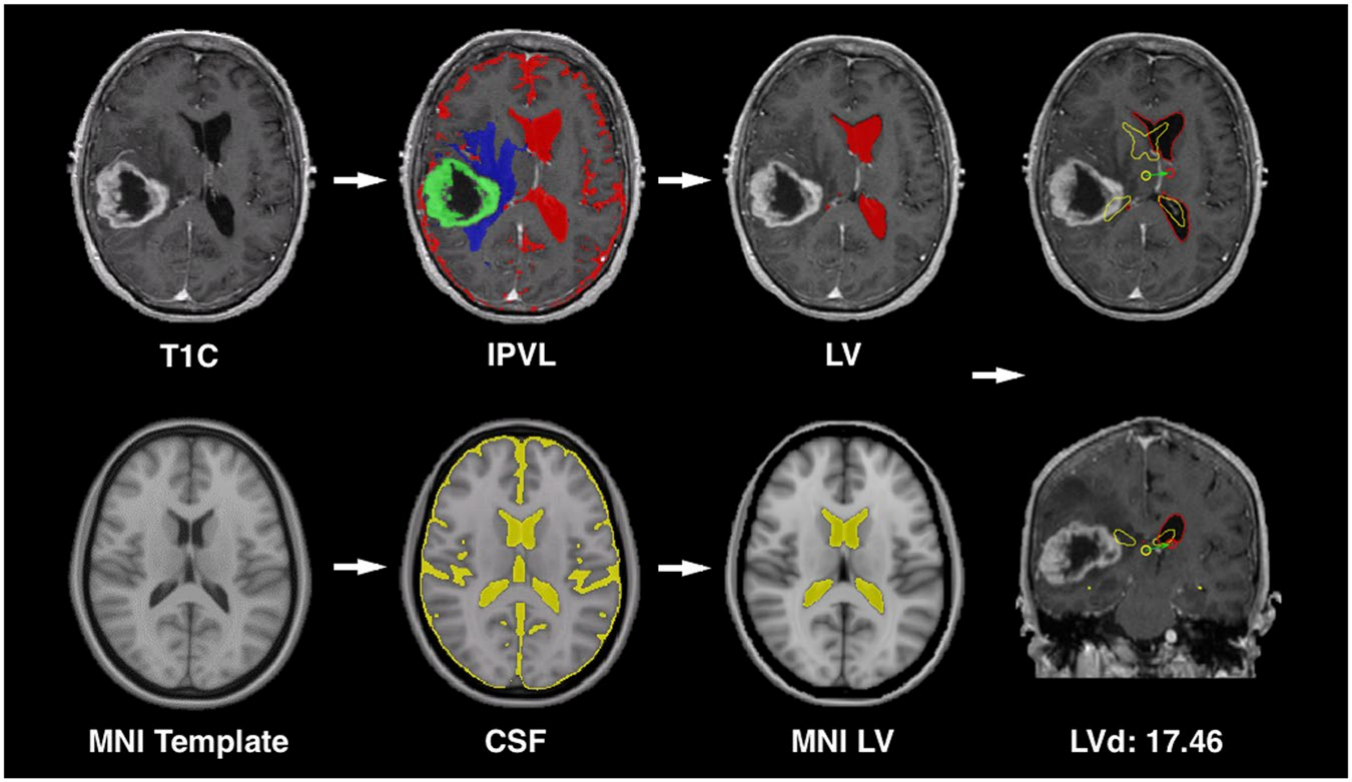
Workflow for generation of Lateral Ventricular Displacement. Preprocessed images were registered to the Montreal Neurological Institute (MNI) template and segmented according to the IPVL pipeline. The magnitude of the vector of displacement of the lateral ventricular segmentation volume (LV red) is calculated relative to the MNI ventricular volume (MNI LV yellow).

Using automatic methods of segmentation developed by our laboratory^8,9^, we propose a novel radiographic parameter for quantifying mass effect related to glioblastoma, which we termed lateral ventricle displacement (LVd). LVd measures the magnitude of displacement from the center of mass of the lateral ventricle volume in glioblastoma patients relative to the center of mass of the lateral ventricle volume from the standard Montreal Neurological Institute (MNI) template brain (Fig. 1). We demonstrated that LVd was significantly elevated in glioblastoma patients relative to subjects without diagnosis of brain tumor. Moreover, increased LVd in patients with glioblastoma was closely associated with reduced clinical survival. Finally, elevated LVd in glioblastoma is associated with increased expression of genes related to increased cellular proliferation, while tumors with low levels of LVd expressed genes involved in cell migration and motility.

## Methods

### Data and image acquisition

Pre-operative MR images from 214 glioblastoma patients from The Cancer Imaging Archive (TCIA) glioblastoma cohort (http://cancerimagingarchive.net) and 550 non-tumor control subjects obtained from the Information eXtraction from Images (IXI) cohort from the Biomedical Image Analysis Group, Imperial College London (http://brain-development.org/ixi-dataset/) were used in this study. Inclusion criteria included patients with at least one artifact free pre-operative T1 weighted MR image with contrast. Patient demographic data is listed in Table 1. In addition to patient clinical data, Level 3 probe collapsed Messenger RNA (mRNA) expression data (Affymetrix HT HG U133A array) for a subset of 199 patients and Level 3 mRNA Sequencing data for a subset of 44 patients were downloaded from the TCGA Data Portal (https://tcga-data.nci.nih.gov/docs/publications/tcga/, https://portal.gdc.cancer.gov/).

**Table 1:** Table of demographics and available data for the cohort studied.

Variable	
N (%)	214 (100)
Age (yrs), N (%), mean ± SD	214 (100), 59.6 ± 13.8
Sex	
Male, N (%)	128 (59.8)
Female, N (%)	86 (40.2)
Race, N (%)	
Caucasian	188 (87.9)
African – American or Black	10 (4.7)
Asian	5 (2.3)
Hispanic or Latino	3 (1.4)
Not Hispanic or Latino	2 (0.9)
Unknown	6 (2.8)
KPS, N (%), mean ± SD	179 (83.6), 77.5 ± 14.1
OS (days), N (%), mean ± SD	209 (97.7), 368.7 ± 337.9
PFS (days), N (%), mean ± SD	200 (93.5), 261.2 ± 271.8
CEV, N (%), mean ± SD	214 (100%), 32492 ± 23904
FHV, N (%), mean ± SD	143 (66.8%), 86991 ± 51684
LVd, N (%), mean ± SD	214 (100), 9.4 ± 4.9
Subtype, N (%)	
Proneural	45 (21.0)
Neural	39 (18.2)
Classical	51 (23.8)
Mesenchymal	57 (26.6)
G-CIMP	9 (4.2)
Unknown	13 (6.1)
U133 mRNA data N (%)	199 (93.0)
mRNAseq data N (%)	44 (20.6)

CE: contrast enhancing; SD: standard deviation; KPS: Karnofsky Performance Scale; OS: overall survival; PFS: progression-free survival; CEV: contrast-enhancing volume; FHV: FLAIR hyperintensity volume; LVd: lateral ventricle displacement.

### Image preprocessing, registration, and segmentation

Spatial and intensity distortions caused by nonlinearity warping were corrected using previously described methods^10^. In order to compare LVd across all subjects, all images were registered to the Montreal Neurological Institute (MNI) 152 nonlinear 1 mm^3^ template using Advanced Normalization Tools (ANTS)^11^. CEV and FHV of each glioblastoma patient was segmented using our previously published iterative probabilistic voxel labeling (IPVL) segmentation algorithm^8^. Previously we demonstrated that IPVL contrast enhancing volumes were statistically indistinguishable from volumes generated by expert operators across all subjects (*P* = 0.93).

While CE and FH volumes can be quite challenging to segment reliably, CSF volumes are more easily derived from imaging given their more uniform appearance and defined MR imaging characteristics T1 (dark) and T2 (bright). To ensure accuracy in this study, all LV segmentations and deformable registrations to MNI template were manually reviewed by three independent reviewers (T.C.S, J.M.T, K.S.P) after each step to ensure preprocessing was successful and accurate for all subjects.

### Lateral Ventricular Displacement

To automate the calculation of LVd, two extra procedures were utilized during image segmentation. Using ANTS^12–14^, a widely studied neuroimaging toolkit for image registration and normalization, non-linear diffeomorphic registration was performed which defined a warp field to apply to the standard lateral ventricle (LV) segmentation^15^. The warp field when applied to the standard LV segmentation would allow for segmentation of the LVs in each tumor subject and was further masked to exclude regions of tumor pathology. The resulting LV segmentation center of mass was calculated. The magnitude of the displacement vector from the subject’s LV center of mass and the template’s LV center of mass was defined as the LVd. An illustrative example of LVd derivation for a subject is shown in (Fig. 1). All comparisons were performed in the common MNI template space.

### Survival Analysis

Quantitative radiographic parameters were analyzed with respect to overall patient survival. A Kaplan-Meier survival curve was generated using a median cutoff of LVd. Cox regression analysis was also performed with respect to age and KPS. All survival analyses were performed using the statistics software SPSS (IBM Corp. Released 2011. IBM SPSS Statistics for Windows, Version 20.0. Armonk, NY: IBM Corp).

### Differential Expression Analysis

Differential gene expression was performed using the available mRNA expression data and mRNAseq data from the TCGA data portal. The groups were dichotomized using the median of LVd as a cutoff. 10,000 cycles of permutation testing and bootstrapping using random sampling with replacement were applied during each analysis. Initially 12, 042 genes were considered in the study. All genes identified by differential expression analysis were corrected for multiple comparisons by Benjamini-Hochberg correction. Gene ontology analysis was performed using DAVID, (https://david.ncifcrf.gov/), and the Gene Ontology Consortium (http://www.geneontology.org/)^16^.

### Data Availability

The datasets generated and analyzed during the current study are available from the corresponding author upon reasonable request.

## Results

**Figure 2:**
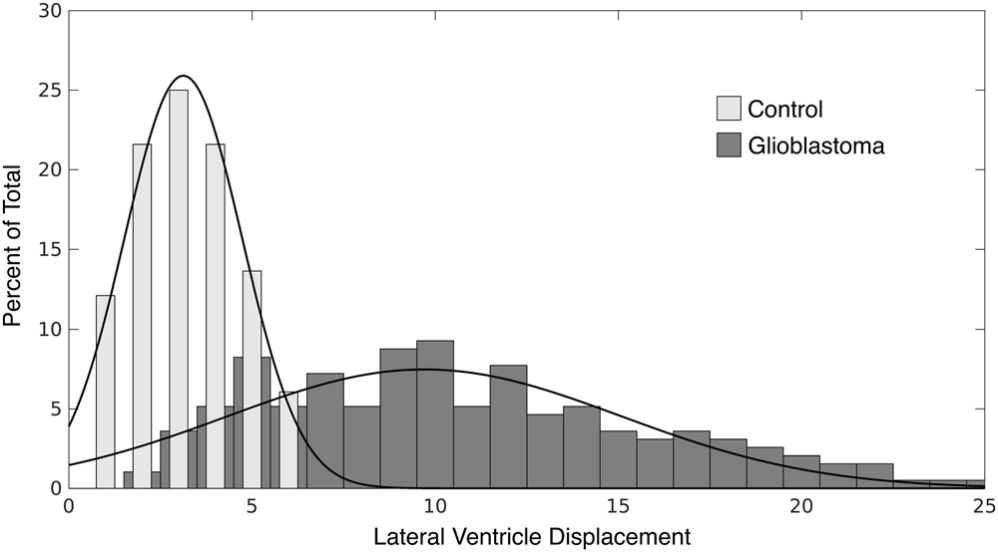
LVd calculations were performed on 550 “normal” (control light grey) MRI’s obtained from the non-tumor control subjects from the University College in London IXI cohort and compared to LVd measurements obtained from glioblastoma subjects (Glioblastoma dark grey).

LVd was determined for 550 subjects without diagnosis of brain tumor from the IXI cohort as well as 214 TCIA glioblastoma subjects. Demographics for these subjects can be found in Table 1. For the subjects without brain tumor, LVd followed a normal distribution that ranged 0.53 to 6.46, with a mean of 3.45 and a standard deviation (SD) of 1.70 (Fig. 2).

For the TCIA glioblastoma patients, significantly higher LVd was observed. The distribution of LVd ranged 1.88 to 29.11 mm, mean LVd of 9.49 with a SD of 4.75. This distribution significantly differed from that observed in the cohort of patients without brain tumors (p < 0.001). Notably, 67% of glioblastoma patients exhibited an LVd exceeding that observed in the control imaging cohort.

### Correlation between CEV, FHV, and LVd

**Figure 3:**
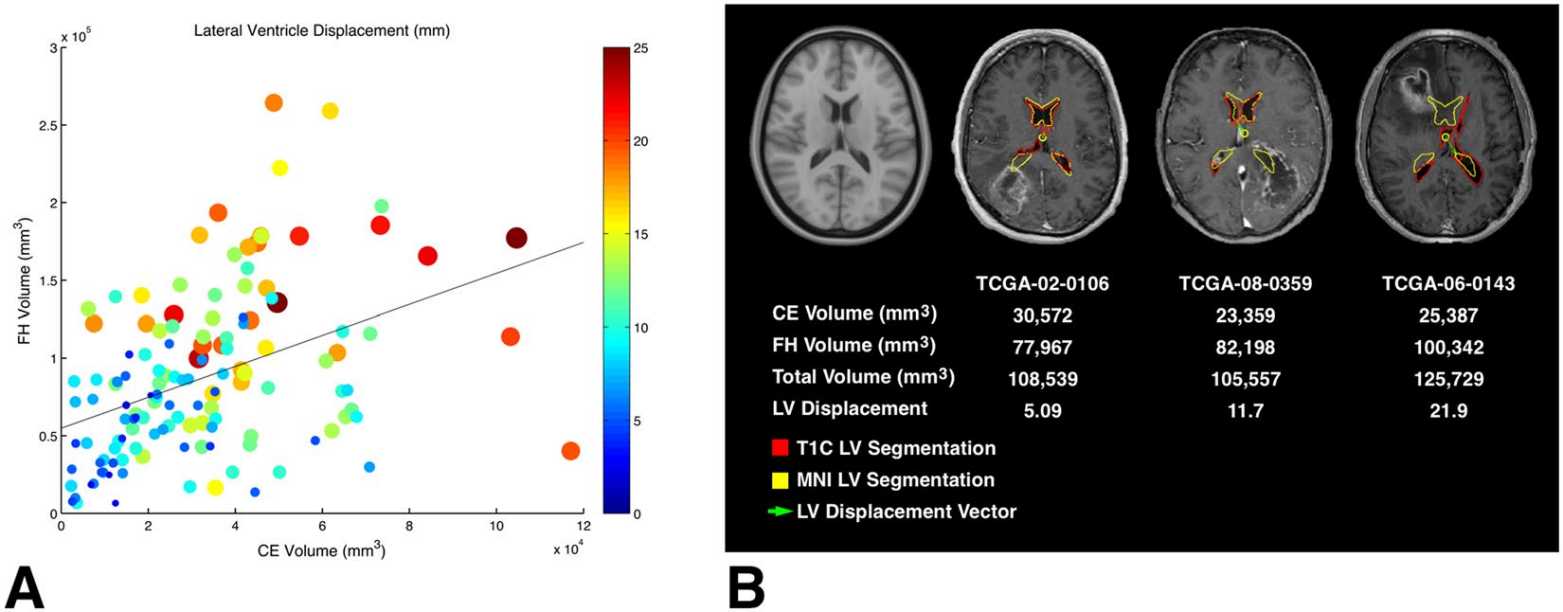
Lateral ventricular segmentation volume is demonstrated in red while the MNI standard CSF volume is in yellow. The vector of displacement is indicated by a green arrow. Notice that LVd varies broadly in these three subjects despite having similar total tumor volumes.

CEV and FHV were segmented for all patients using IPVL. There was a statistically significant correlation between CEV and LVd (R^2^ = 0.32, p < 0.001) as well CEV plus FHV and LVd (R^2^ = 0.53, p < 0.001). Despite this correlation many tumors with similar CEV and FHV vary with respect to LVd (Fig. 3A). (Figure 3B) provides three illustrative examples of patients with tumors of comparable tumor volume which nevertheless exhibited significant variations in LVd. CEV alone accounts for31.7% of the variance in LVd while FHV alone accounts for 21.2% of the variance in LVd. These results suggest that while CEV and FHV indeed contribute to LVd, LVd captures additional information which may better represent physiological processes like the rate of tumor growth and cerebral compliance.

### Association of LVd and glioblastoma survival

**Figure 4:**
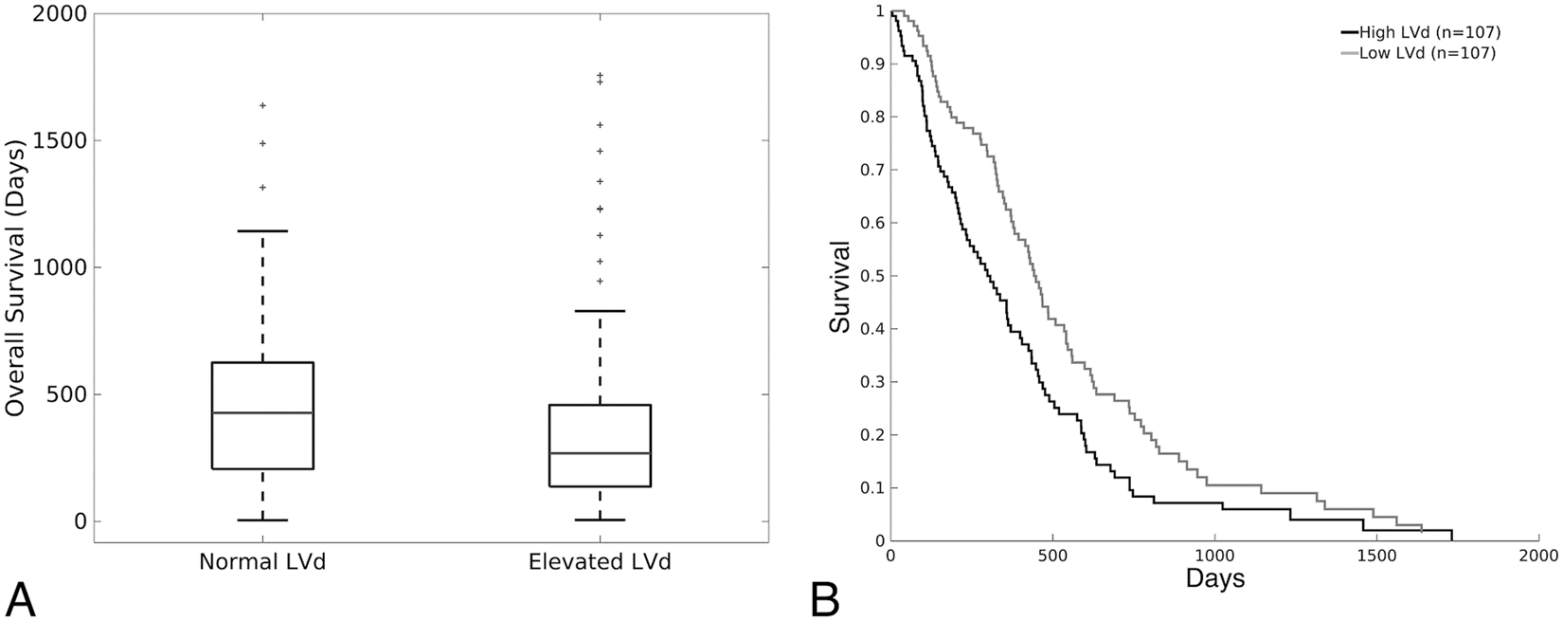
(**A**) Box plot demonstrating survival difference (days of survival) in glioblastoma patients with “normal” versus elevated LVd. (**B**) Kaplan Meier survival plot by median cut-off of LVd in the cohort. Demonstrates prolonged survival in glioblastoma patients with low LVd (gray). High LVd is indicated in black.

We observed that the 33% of glioblastoma patients with LVd within the ranges of control subjects (0.53 to 6.46 mm) survived >150 days longer than patients with LVd greater than this range (median of 268 days vs. 427 days, p > 0.001) (Fig. 4A). When the glioblastoma cohort was dichotomized by the median value of LVd, Kaplan-Meier survival analysis revealed that high LVd was strongly associated with reduced overall patient survival. (Log Rank p = 0.004, n = 214, Fig. 4B). This finding is largely consistent with previous studies demonstrating prognostic value for qualitative measures of mass effect in glioblastoma patients^17–22^.

To further investigate the relationship between LVd and clinical survival, we performed a multivariate Cox regression analysis of survival as a function of LVd using the TCIA dataset. This analysis confirmed that LVd is associated with reduced patient survival (p = 0.021, n = 214). When patient age, a known survival-associated measure, was added to the Cox regression model, LVd remained significantly associated with reduced patient survival (p = 0.012, n = 214). Similarly, when patient Karnofsky Performance Score (KPS) was added to the Cox regression model, LVd remained significantly associated with reduced patient survival (p = 0.032, n = 179 Table 2). These results support LVd as an important potential prognostic marker of glioblastoma survival. CEV was not associated with survival in Cox regression models that accounted for both age and KPS (Table 3).

**Table 2:** Cox regression analysis with respect to LVd, age, and KPS showing hazard ratio, 95% confidence interval (CI), and *p* values. Data indicated with subscript alone show data for univariate analysis.

	Hazard Ratio	95% CI	*p* value
OS^a^			
LVd	1.033964	1.002828–1.066067	0.0321
Age	1.024188	1.009242–1.039355	0.0013
KPS	0.976481	0.963175–0.989971	0.0006
LVd	1.035723	1.007492–1.064746	0.0124
Age	1.020303	1.018941–1.045647	<0.001
LVd_alone_	1.033551	1.005575–1.062304	0.0183
PFS			
LVd	1.033861	1.000568–1.068261	0.0458
Age	1.015519	1.000700–1.030558	0.0409
KPS	0.987677	0.974982–1.000536	0.0576
LVd	1.038004	1.008326–1.068556	0.0117
Age	1.020303	1.007585–1.033183	0.0018
OS (median cut-off)			
LVd > median^b^	1.590741	1.141085–2.217588	0.0062
Age	1.025213	1.010252–1.040395	0.0009
KPS	0.976579	0.963460–0.989876	0.0006
LVd > median_alone_	1.584391	1.177574–2.131752	0.0024

^a^Abbreviations: LVd: lateral ventricle displacement, OS: overall survival, PFS: progression-free survival, LKF: last known follow-up.

^b^Median is 8.5921.

**Table 3:** Cox regression analysis with respect to CEV, age, and KPS showing hazard ratio, 95% confidence interval (CI), and *p* value.

	Hazard Ratio	95% CI	*p* value
OS^a^			
CEV	1.006018	0.998553–1.013539	0.1110
Age	1.024495	1.009347–1.039870	0.0014
KPS	0.976383	0.963268–0.989678	0.0006
PFS			
CEV	1.009545	1.001858–1.017292	0.0152
Age	1.016129	1.001105–1.031378	0.0350
KPS	0.987874	0.975177–1.000736	0.0637
OS (median cut-off)			
CEV > median^b^	1.374789	0.989468–1.910162	0.0578
Age	1.023983	1.009041–1.039147	0.0017
KPS	0.975798	0.962690–0.989084	0.0004

^a^Abbreviations: CEV: contrast-enhancing volume, OS: overall survival, PFS: progression-free survival, LKF: last known follow-up.

^b^Median is 29, 151 mm^3^.

Mass effect is often estimated clinically by measuring midline shift, which has gained popularity due to its ease of calculation and the prevalence and familiarity of axial imaging. This metric however, only encompasses the degree of mass effect in the left-right (x-axis) plane and is susceptible to significant inter-operator variability. Additionally, the metric is best suited to measure the mass effect by laterally located tumors and may underestimate the mass effect of tumors situated anteriorly, posteriorly, inferiorly, or superiorly where the predominant mass effect vector would be in the anterior-posterior or inferior-superior direction. To examine whether x-axis lateral ventricle displacement (LVx), a proxy for conventional midline shift, demonstrated the same survival association of LVd we performed cox regression analyses between the LVx and survival with and without covarying for KPS and age. As expected, we found that LVd was positively correlated with LVx (r = 0.875, p = < 0.001). Additionally, we found that LVx was associated with survival (p < 0.01) but no longer met statistical significance after correcting for age and KPS (p = 0.09). As LVx only accounts for one of the vectors of the three cardinal directions, it is only a partial estimation of the force exerted by the tumor on the surrounding parenchyma where as LVd may be a better proxy of mass effect.

### Gene expression pattern as a function of LVd

**Figure 5:**
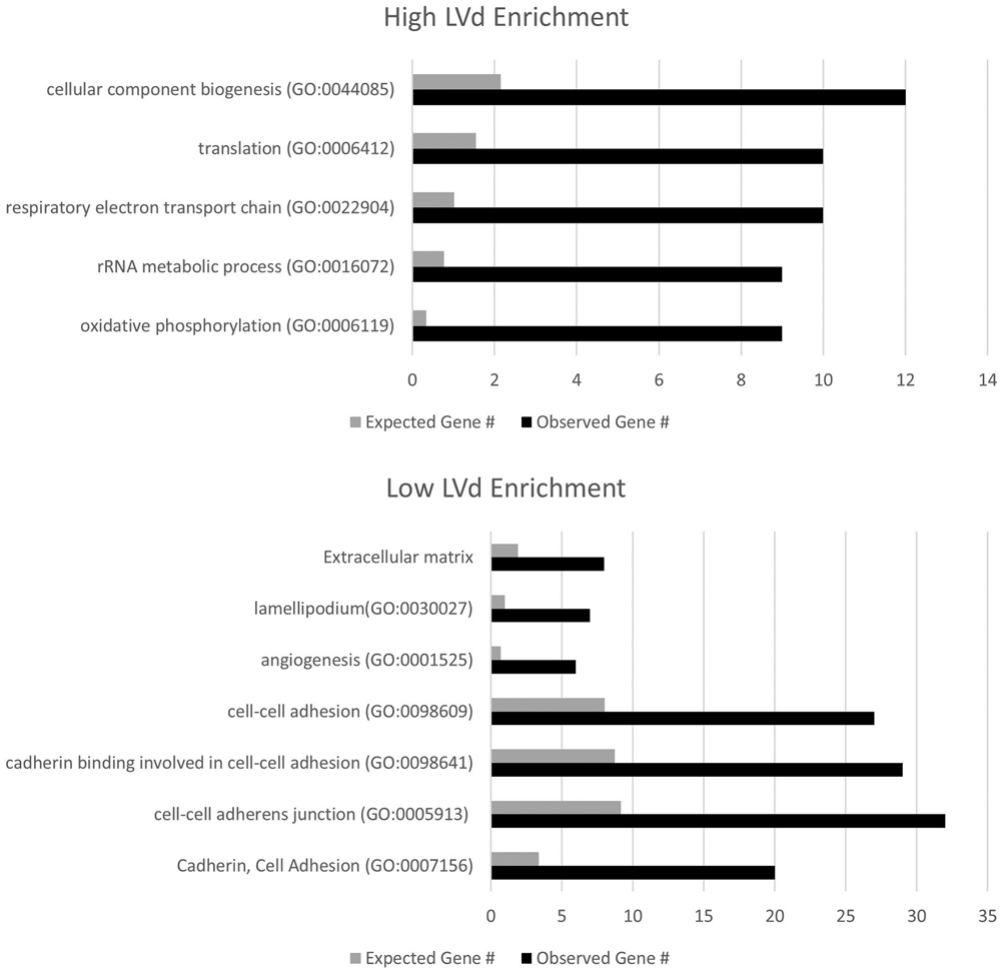
Box plot of high and low LVd associated genetic enrichment for significant Gene Ontology (GO) terms.

To better understand the biology that underlies differential LVd, expression analyses were performed (See Methods). Genes that were conserved in both the mRNA and mRNAseq were fed into gene ontology and functional annotation data sets (See Methods). The analyses indicated that glioblastomas with higher LVd expressed gene signatures associated with cell growth, including genes required for translation, mitochondrial metabolism, cellular component biogenesis, and oxidative phosphorylation (Fig. 5a). In contrast, gene signatures associated with invasion, including those required for cell adhesion, cell migration, motility, and angiogenesis were expressed in glioblastoma with low LVd (Fig. 5b).

## Discussion

The concept of mass effect plays a central role in defining the premise for justifying surgery of the cerebrum^23–25^. In neurologic diseases, ranging from trauma^23,26^, stroke^21,24^, to tumor^20^, increased mass effect is consistently associated with poor prognosis. Glioblastoma is no exception. Increased mass effect on pre-surgical MRIs of glioblastoma patients is associated with poor prognosis^17–22^. In clinical practice, mass effect is typically characterized by terms such as midline shift or various forms of herniation syndromes^27^. These qualitative terms lack the rigor of objective quantification that ensures precision and reproducibility. For instance, the plane on MR image through which midline shift is defined and how midline shift is measured vary from observer to observer. Here, we utilized a previously validated segmentation algorithm^8,9^ to describe LVd as a novel radiographic measure that allows automated, quantitative assessment of mass effect. The basic concept is to determine the totality of shift in cerebrospinal fluid volume as a proxy for mass effect. We characterized the normal range of variability in this parameter in 550 patients without diagnosis of brain tumor and demonstrate that glioblastoma patients exhibited elevated LVd. Importantly, the magnitude of LVd in glioblastoma patients robustly associated with overall survival, after accounting for pertinent clinical variables, suggesting its potential utility as a prognostic imaging biomarker.

The consistency of mass effect as a glioblastoma prognostic factor in published reports^17–22^ contrasts to the conflicting literature of CEV and FHV in this regard^28–30^. One interpretation of this discrepancy is that while the CEV and FHV provide information pertaining to tumor burden, robust survival prognostication requires integration of these variables and the capacity of the cerebrum to compensate. Unfortunately, the compliance of the human cerebrum *in vivo* remains poorly characterized^31–33^. Pertaining to this matter, it is notable that tumors of comparable CEV and FHV can be associated with a wide range of LVd (Fig. 3B).

Correlative analysis between LVd and glioblastoma genomic profile provides a window into the tumor biologic processes that contribute to mass effect. This analysis suggests that LVd (hence, mass effect) represents an integrative measure of the proliferative potential and the invasive potential of the tumor. The counterbalance between these two potentials (Fig. 5) is reminiscent of the “go or grow” hypothesis, where cell fate is predominantly committed to either migration or proliferation^34^. Our genomic analysis suggests that glioblastomas in which the prevailing cell population are committed to proliferation are more likely to be associated with increased mass effect. In contrast, glioblastomas in which most cells are committed to migration are less likely to cause mass effect. In this regard, LVd may harbor predictive value for therapeutics targeting proliferative or invasive processes.

From a translational perspective, LVd is an attractive parameter since it can be reliably and reproducibly calculated in a timely manner as to facilitate integration into a clinical work flow. That said, issues pertaining to LVd interpretation require discussion. While our study represents 214 subjects from the TCIA, which includes multiple different sites around the country, we hope to see further validation in glioblastoma cohorts to confirm its findings. Since LVd is calculated relative to the MNI template, normal anatomic variations or intrinsic asymmetry in ventricular anatomy will impact LVd calculation. As such, the LVd value in any tumor subject will need to be interpreted in the context of the distribution of LVd that we characterized in 550 non-tumor patients (Fig. 2). Additionally, because LVd is determined from the magnitude of a 3 dimensional vector of displacement, some components (directions) of this vector may be more informative than others. Future directions of our research will aim to further elucidate the nature of mass effect and further characterize the relationship of lateral ventricular displacement with known mass effect measures. Finally, the calculation of LVd will inevitably be influenced by the quality of the MR, and standardization of MR sequences will be needed for cross comparison of LVd values between institutions and scanners. We attempted to address this limitation by employing IPVL automated segmentation to the data set that was designed to handle heterogeneous datasets, and carefully reviewed all steps of or processing through image segmentation to ensure both accuracy and quality of our results. Future directions of our research will aim to further elucidate the nature of mass effect and further characterize the relationship of lateral ventricular displacement with known mass effect measures.

Finally, the calculation of LVd will inevitably be influenced by the quality of the MR, and standardization of MR sequences will be needed for cross comparison of LVd values between institutions and scanners. We addressed this limitation by employing IPVL, a validated, automated segmentation algorithm^8,9^ designed to handle heterogeneous datasets. Moreover, the resultant segmentation of the CSF space was carefully reviewed independently by T.S., J.T., M.B., and K.P to ensure both accuracy and quality.

In sum, we provide data supporting the utility of LVd as a tool for quantitating glioblastoma related mass effect. We further explore the biologic processes within glioblastoma that associate with LVd. To the extent that mass effect bears prognostic value in other neurologic diseases^20,21,23,24,26^, clinical application of this imaging biomarker likely extends beyond glioblastoma.

## References

[1] Ostrom QT (2015). CBTRUS Statistical Report: Primary Brain and Central Nervous System Tumors Diagnosed in the United States in 2008-2012. Neuro Oncol.

[2] Shiroishi MS (2013). Posttreatment evaluation of central nervous system gliomas. Magn Reson Imaging Clin N Am.

[3] Chaddad A, Desrosiers C, Hassan L, Tanougast C (2016). A quantitative study of shape descriptors from glioblastoma multiforme phenotypes for predicting survival outcome. Br J Radiol.

[4] Macdonald DR, Cascino TL, Schold SC (1990). & Cairncross, J. G. Response criteria for phase II studies of supratentorial malignant glioma. J Clin Oncol.

[5] Wen PY (2010). Updated response assessment criteria for high-grade gliomas: response assessment in neuro-oncology working group. J Clin Oncol.

[6] Mokri B (2001). The Monro-Kellie hypothesis: applications in CSF volume depletion. Neurology.

[7] Ropper AH (1986). Lateral displacement of the brain and level of consciousness in patients with an acute hemispheral mass. N Engl J Med.

[8] Steed TC (2015). Iterative probabilistic voxel labeling: automated segmentation for analysis of The Cancer Imaging Archive glioblastoma images. AJNR Am J Neuroradiol.

[9] Steed, T. C. *et al*. Differential localization of glioblastoma subtype: implications on glioblastoma pathogenesis. *Oncotarget*, 10.18632/oncotarget.8551 (2016). 10.18632/oncotarget.8551 PMC5041878 27056901

[10] Jovicich J (2006). Reliability in multi-site structural MRI studies: effects of gradient non-linearity correction on phantom and human data. NeuroImage.

[11] Jenkinson M, Bannister P, Brady M, Smith S (2002). Improved optimization for the robust and accurate linear registration and motion correction of brain images. Neuroimage.

[12] Abraham A (2017). Deriving reproducible biomarkers from multi-site resting-state data: An Autism-based example. Neuroimage.

[13] Schlichting ML, Mumford JA, Preston AR (2015). Learning-related representational changes reveal dissociable integration and separation signatures in the hippocampus and prefrontal cortex. Nat Commun.

[14] Maass A (2014). Laminar activity in the hippocampus and entorhinal cortex related to novelty and episodic encoding. Nat Commun.

[15] Avants BB (2014). The Insight ToolKit image registration framework. Front Neuroinform.

[16] Ashburner M (2000). Gene ontology: tool for the unification of biology. The Gene Ontology Consortium. Nat Genet.

[17] Li GZ, Yang J, Ye CZ, Geng DY (2006). Degree prediction of malignancy in brain glioma using support vector machines. Comput Biol Med.

[18] Lacroix M (2001). A multivariate analysis of 416 patients with glioblastoma multiforme: prognosis, extent of resection, and survival. J Neurosurg.

[19] Zacharaki EI, Hogea CS, Shen D, Biros G, Davatzikos C (2009). Non-diffeomorphic registration of brain tumor images by simulating tissue loss and tumor growth. Neuroimage.

[20] Gamburg ES (2000). The prognostic significance of midline shift at presentation on survival in patients with glioblastoma multiforme. Int J Radiat Oncol Biol Phys.

[21] Zazulia AR, Diringer MN, Derdeyn CP, Powers WJ (1999). Progression of mass effect after intracerebral hemorrhage. Stroke.

[22] CO DAF (2013). Long-term outcome in patients with recurrent malignant glioma treated with Perillyl alcohol inhalation. Anticancer Res.

[23] Bullock, M. R. *et al*. Surgical management of traumatic parenchymal lesions. *Neurosurgery* **58**, S25-46, discussion Si-iv, 10.1227/01.NEU.0000210365.36914.E3 (2006). 10.1227/01.NEU.0000210365.36914.E3 16540746

[24] McKenna A, Wilson CF, Caldwell SB, Curran D (2012). Functional outcomes of decompressive hemicraniectomy following malignant middle cerebral artery infarctions: a systematic review. Br J Neurosurg.

[25] Gonda DD (2013). The value of extended glioblastoma resection: Insights from randomized controlled trials. Surg Neurol Int.

[26] Kim JJ, Gean AD (2011). Imaging for the diagnosis and management of traumatic brain injury. Neurotherapeutics.

[27] Mariano GL, F. M., Hoffman C, Rosengart A. in *Principles of Critical* Care (ed. Schmidt, G.A. Hall, J.B. & Kress, J.P.) (McGraw-Hill, 2014).

[28] Grossman R (2017). Dynamics of FLAIR Volume Changes in Glioblastoma and Prediction of Survival. Ann Surg Oncol.

[29] Li WB (2012). MRI manifestions correlate with survival of glioblastoma multiforme patients. Cancer Biol Med.

[30] Zhang Z (2014). Identifying the survival subtypes of glioblastoma by quantitative volumetric analysis of MRI. Journal of neuro-oncology.

[31] Howells T, Lewen A, Skold MK, Ronne-Engstrom E, Enblad P (2012). An evaluation of three measures of intracranial compliance in traumatic brain injury patients. Intensive Care Med.

[32] Raboel PH, Bartek J, Andresen M, Bellander BM, Romner B (2012). Intracranial Pressure Monitoring: Invasive versus Non-Invasive Methods-A Review. Crit Care Res Pract.

[33] Tain RW, Alperin N (2009). Noninvasive intracranial compliance from MRI-based measurements of transcranial blood and CSF flows: indirect versus direct approach. IEEE Trans Biomed Eng.

[34] Hatzikirou H, Basanta D, Simon M, Schaller K, Deutsch A (2012). ‘Go or grow’: the key to the emergence of invasion in tumour progression?. Math Med Biol.

